# Transcription factor FoxO1 regulates myoepithelial cell diversity and growth

**DOI:** 10.1038/s41598-024-51619-1

**Published:** 2024-01-11

**Authors:** Rino Tokumasu, Rika Yasuhara, Seya Kang, Takahiro Funatsu, Kenji Mishima

**Affiliations:** 1 Division of Pathology, Department of Oral Diagnostic Sciences, School of Dentistry, Showa University, Tokyo, 142-8555 Japan; 2 Division of Dentistry for Persons with Disabilities, Department of Perioperative Medicine, Graduate School of Dentistry, Showa University, Tokyo, 142-8555 Japan; 3 Division of Dentistry for Persons with Disabilities, Department of Perioperative Medicine, School of Dentistry, Showa University, Tokyo, 142-8555 Japan; 4 Department of Pediatric Dentistry, School of Dentistry, Showa University, Tokyo, 142-8555 Japan

**Keywords:** Differentiation, Cell lineage

## Abstract

Salivary gland myoepithelial cells regulate saliva secretion and have been implicated in the histological diversity of salivary gland tumors. However, detailed functional analysis of myoepithelial cells has not been determined owing to the few of the specific marker to isolate them. We isolated myoepithelial cells from the submandibular glands of adult mice using the epithelial marker EpCAM and the cell adhesion molecule CD49f as indicators and found predominant expression of the transcription factor FoxO1 in these cells. RNA-sequence analysis revealed that the expression of cell cycle regulators was negatively regulated in FoxO1-overexpressing cells. Chromatin immunoprecipitation analysis showed that FoxO1 bound to the p21/p27 promoter DNA, indicating that FoxO1 suppresses cell proliferation through these factors. In addition, FoxO1 induced the expression of ectodysplasin A (Eda) and its receptor Eda2r, which are known to be associated with X-linked hypohidrotic ectodermal dysplasia and are involved in salivary gland development in myoepithelial cells. FoxO1 inhibitors suppressed Eda/Eda2r expression and salivary gland development in primordial organ cultures after mesenchymal removal. Although mesenchymal cells are considered a source of Eda, myoepithelial cells might be one of the resources of Eda. These results suggest that FoxO1 regulates myoepithelial cell proliferation and Eda secretion during salivary gland development in myoepithelial cells.

## Introduction

Myoepithelial (ME) cells surround secretory units to maintain apical basal polarity and stimulate fluid secretion in exocrine glands such as the mammary and salivary glands^1,2^. In addition to their basic features, ME cells are thought to be involved in cell diversity in salivary glands^3,4^. Although ME cells lack stem-like features during normal development^5,6^, they have been reported to express several stem cell markers^7–11^, and transformed into ductal structures in three-dimensional sphere culture^12^. In particular, histological diversity constructed by various epithelial and mesenchymal differentiations is observed in ME cell-originated salivary gland tumors, such as pleomorphic adenomas^13,14^. However, the precise molecular mechanism that determines ME cell characteristics and functions remain unknown.

Several cell surface markers have been used in our study and in other studies to isolate ME cells from tissues^15–17^. The combination of an epithelial cell adhesion molecule (EpCAM) and alpha 6 integrin (CD49f) enabled the isolation of a particular cell type^12,17,18^. We previously found that ME cells with low EpCAM but high CD49f expression, which were isolated by flow cytometry, express FoxO1 as well as ME markers, including alpha smooth muscle actin (αSMA)^12^. FoxO1 belongs to the forkhead box (FOX/FKHR) transcription factor family, which consists of four members (FoxO-1, -3, -4, and -6), and binds to the daf-16 domain (TTGTTTA) to transactivate target genes, such as p21 and p27^19,20^. Akt, is downstream of the insulin/PI3K pathway and inactivates FoxO1 by phosphorylation, causing FoxO1 translocation from the nucleus to cytoplasm^20,21^. LPS and NF-κB signaling have a dual effect on FoxO1 depending on the microenvironment^22–24^. NF-κB acts as a negative feedback for FoxO1, when insulin signaling is activated or FoxO1 is acetylated and is sensitive to phosphorylation^22,25,26^. However, FoxO1 enhances NF-κB-binding to its response elements and synergistically amplifies NF-κB activity^23,24,27^. FoxO1 also contributes to tissue-specific cell fate decisions via NF-κB in pancreatic β-cells, adipocytes, endometrial stromal cells, and macrophage^20,24,28,29^. FoxO1 acts as a transcription factor that binds to the daf16 response element, which express the target genes and induces several biological effects via NF-κB activation^21,27^.

FoxO1 has a wide range of functions, including regulation of the cell cycle in the pancreas^28^ and vascular endothelial cells^30^, differentiation of adipocytes^20,31^ and muscle cells^32^, apoptosis, insulin-dependent energy metabolism^21^, and oxidative stress resistance^33,34^ in various cells. Although FoxO1 is ubiquitously expressed, it is thought to have tissue-specific functions^26,34^. Downregulation of FoxO1 in the salivary glands were reported in Sjögren’s syndrome, which is characterized by a severe hypofunction of salivary glands suggests its involvement in the autoimmune response and exocrine cell death^35–37^. However, there have been few reports on the expression and function of FoxO1 in the salivary glands. Therefore, the present study investigated the function of FoxO1 in salivary ME cells.

## Results

### FoxO1 is predominantly expressed in ME cells

**Figure 1:**
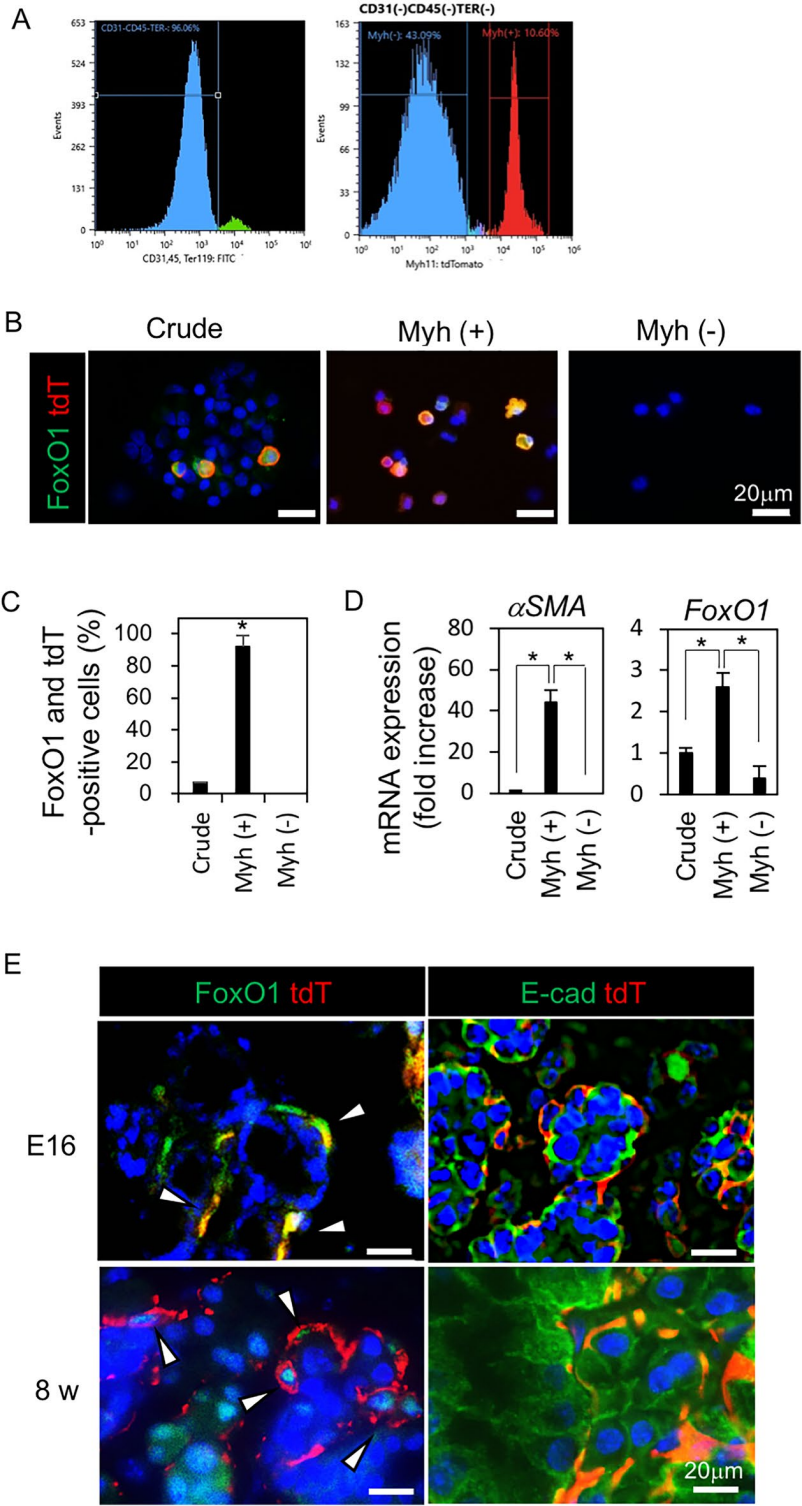
FoxO1 was predominantly expressed in ME cells. (**A–D**) ME cells were isolated from 8-week-old male Myh11-CreER^T2^/tdTomato (tdT)^fl/fl^ mice (n = 4). Cre recombination was induced by tamoxifen injection 1 day before the assay. (**A**) Flow cytometry histograms. The Myh11-positive ME cells (tdT^+^) represented 10.6% (right) of salivary gland tissue cells without endothelial cells, hematocytes, and erythroid cells (CD31^−^C45^−^TER119^−^; 96.06%, left). (**B,C**) tdT fluorescence and FoxO1 immunofluorescence (IF) in FACS-sorted cells [crude, Myh(+), Myh(−)] (**B**). Bar = 20 μm. The cell population expressing FoxO1 and tdT double positive (%) in each FACS-sorted cells [crude, Myh(+), Myh(−)] (**C**). Threshold intensity was 30. *P < 0.05. (**D**) Expression of *αSMA* and *FoxO1* in CD31^−^C45^−^TER119^−^ cells (crude), tdT-positive [Myh(+)] and -negative [Myh(−)] cells. *P < 0.05. (**E**) tdT fluorescence and IF of FoxO1 and E-cadherin (E-cad) in submandibular glands (SMG) of Myh11-CreER^T2^/tdT^fl/fl^ on embryonic day 16 (E16, n = 4) and at 8 weeks (8w, n = 3). The arrow head showed αSMA and FoxO1 double positive cells. Bar = 20 μm. All data were representative of three independent experiments. See also Supplementary Fig. S1.

For the adult ME cells labeling, we used CreER^T2^ driver mice under the control of SMMHC/MYH11 (myosin heavy chain 11) promoter, which is suitable for lineage tracing ME cells as the αSMA promoter. ME cells in the submandibular glands (SMGs) were isolated from Myh11-CreER^T2^/tdTomato (tdT)^fl/fl^ mice. In this isolation system, the digested cells contained a small number of αSMA-positive mesenchymal cells such as vascular endothelial or smooth muscle cells and pericytes. To avoid contamination of endothelial-, hematopoietic- and erythroid- cells, CD31-, CD45, and TER119-positive cells were initially eliminated (Fig. 1A, left). ME cells in tamoxifen-treated Myh11-CreER^T2^/tdT^fl/fl^ mice were detected as Myh11-positive cells (tdT+; 10.6%) after elimination of endothelial cells, hematocytes, and erythroid cells (CD31-C45-TER119-; 96.06%), which is the assortment of ME, basal, ductal, acinar, and mesenchymal cells (Fig. 1A, right). ME cells are only located around secretory units and account for approximately 10% of the cells at adult age, which are labeled by Myh11-positive [Myh (+)] tdT fluorescence (Fig. 1E). Immunofluorescence analysis revealed that FoxO1 were contained in 6.9 ± 0.5% of crude cells and 92.1 ± 7% in Myh(+)-sorted cells (Fig. 1B,C). FoxO1 was mainly located in the nucleus (84.3 ± 1.35%) of tdT-positive cells, although expression in the cytoplasm occurs in 16.7 ± 1.35% of the cells. The morphological change of these cells is important, but we cannot point out any difference in cytospin data and tissue sections. Therefore, Further studies are needed to characterize these cells. High expression of *FoxO1* and *αSMA* in Myh (+) cells were also detected by RT-qPCR (Fig. 1D, Fig. S1). FoxO1 expression was detected in the tdT-positive ME cells (Fig. 1E, left) and the tdT-positive ME cells were surrounding by E-cadherin (E-cad)-positive cells in the SMG of Myh11-CreER^T2^/ tdT^fl/fl^ mice on embryonic day 16 (E16) and at 8 weeks (Fig. 1E, right). These results suggested that FoxO1 is predominantly expressed in ME cells.

**Figure 2:**
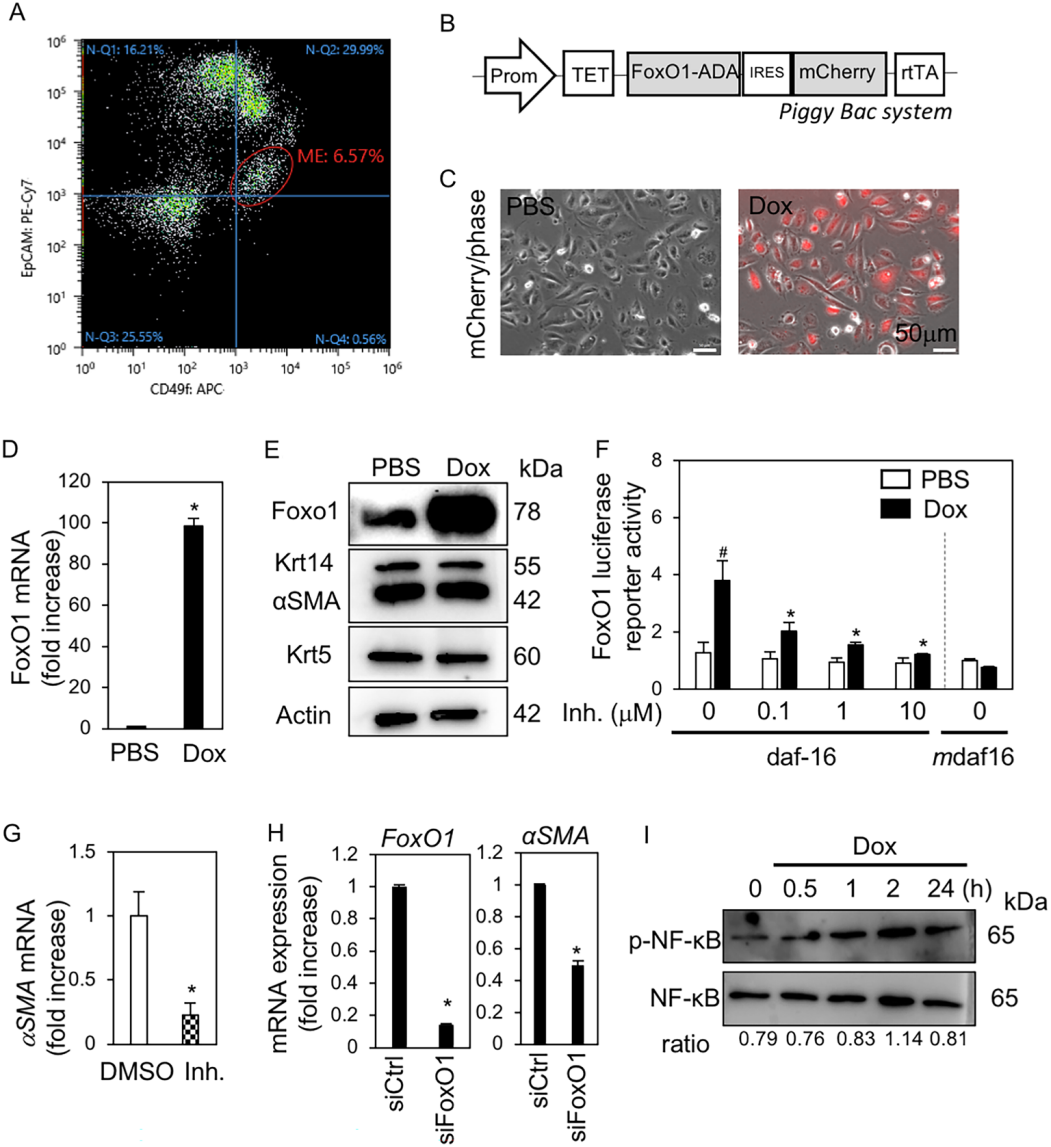
Overexpression of FoxO1 in ME cells. (**A**) Scatter plot of CD49f (x-axis) and EpCAM (y-axis). The cells were isolated from SMG in TP53 mutant female mice (n = 4) and analyzed by flow cytometry. EpCAM^low^CD49f^high^-cells were sorted as ME cells (6.5%). (**B**) A schematic for integration of PiggyBac transposon vector plasmid. The Tet-On inducible gene expression system was used. FoxO1 expression was induced by doxycycline (Dox). (**C**) mCherry fluorescence merged with phase contrast in ME^PB-FoxO1^ cells treated with and without Dox (2 µg/mL) for 48 h. (**D**) Expression of *FoxO1* mRNA in ME^PB-FoxO1^ cells treated with and without Dox for 24 h. *P < 0.05. n = 3. (**E**) Immunoblotting for FoxO1, αSMA, Krt14, Krt5, and β-actin in ME^PB-FoxO1^ cells treated with and without Dox for 72 h. (**F**) FoxO1 luciferase assay in the presence of FoxO1 inhibitor (Inh.; AS1842856) at the indicated concentrations. pGL4 luciferase reporter vector (upper) was constructed to include three FoxO1-binding elements (daf16:TTGTTTA and *m*daf16:TTGCTTA). FoxO1 transcriptional activity was measured. pRL-TK was used as internal control. The Renilla luciferase normalized the firefly luciferase. ^#^*P* < 0.05 vs. control (Ctrl). *P < 0.05 vs. Dox. n = 5. (**G**) Expression of *αSMA* mRNA in ME cells treated with and without FoxO1 inhibitor (Inh.; AS1842856, 1 μM) for 72 h. *P < 0.05. n = 3. (**H**) Expression of *FoxO1* and *αSMA* mRNA in siRNA-mediated knockdown of FoxO1 (siFoxO1) or control (si Ctrl) in ME cells. *P < 0.05. n = 3. (**I**) Immunoblotting for NF-κB/p65 and phospho-NF-κB/p65 in ME^PB-FoxO1^ cells treated with and without Dox at the indicated time-points. The signal intensity of phospho-NF-κB/p65 was normalized to that of NF-κB/p65 (ratio). All data were representative of three independent experiments. See also Supplementary Figs. S2 and S3.

To examine the role of FoxO1 in ME cells, we constructed a tet-on-inducible FoxO1 expression vector using the PiggyBac transposon system (Fig. 2B), and established inducible FoxO1-expressing ME cells (ME^PB-FoxO1^) from TP53 mutant female mice. The EpCAM^low^CD49f^high^-ME cells represented 6.5% of these cells (Fig. 2A). ME^PB-FoxO1^ cell induction with doxycycline (Dox; 2 µg/mL) increased *FoxO1* mRNA expression and mCherry fluorescence (Fig. 2C,D). The protein level of FoxO1 increased after Dox treatment, whereas that of ME markers, such as αSMA, Krt14, and Krt5, did not change (Fig. 2E, Fig. S2). We subsequently measured FoxO1 reporter activity using constructs with FoxO1-binding element (3 × daf16: TTGTTTA) and a mutant (3 × *mutant* daf16:TTGCTTA) in the absence and presence of a FoxO1 inhibitor (Inh.; AS1842856) (Fig. 2F). The Dox-induced FoxO1 reporter activity was approximately four times higher than that of the control, while that of the mutant did not change even after Dox induction (Fig. 2F). AS1842856 (0.1 μM to 10 μM) inhibited FoxO1 reporter activity in a dose-dependent manner. FoxO1 reporter activity was detected at basal levels and AS1842856 inhibited it to 0.6% of basal levels (Fig. S3). Interestingly, AS1842856 (1 μM) strongly inhibited αSMA expression in ME cells (Fig. 2G). Gene silencing of FoxO1 also decreased αSMA expression (Fig. 2H), suggesting that FoxO1 is essential for ME cell properties. Subsequently, we examined NF-κB activation by FoxO1, since FoxO1 expressed several biological functions by promoting NF-κB activation independent of FoxO transcriptional activity. The peak of phospho-NF-κB/p65 up-regulation was observed at 2 h, followed by a decrease 24 h after dox treatment in ME^PB-FoxO1^ cells (Fig. 2I). These results suggest that NF-κB/p65 was enhanced by FoxO1 in ME cells.

### Alteration of FoxO1-mediated transcription in ME cells

**Figure 3:**
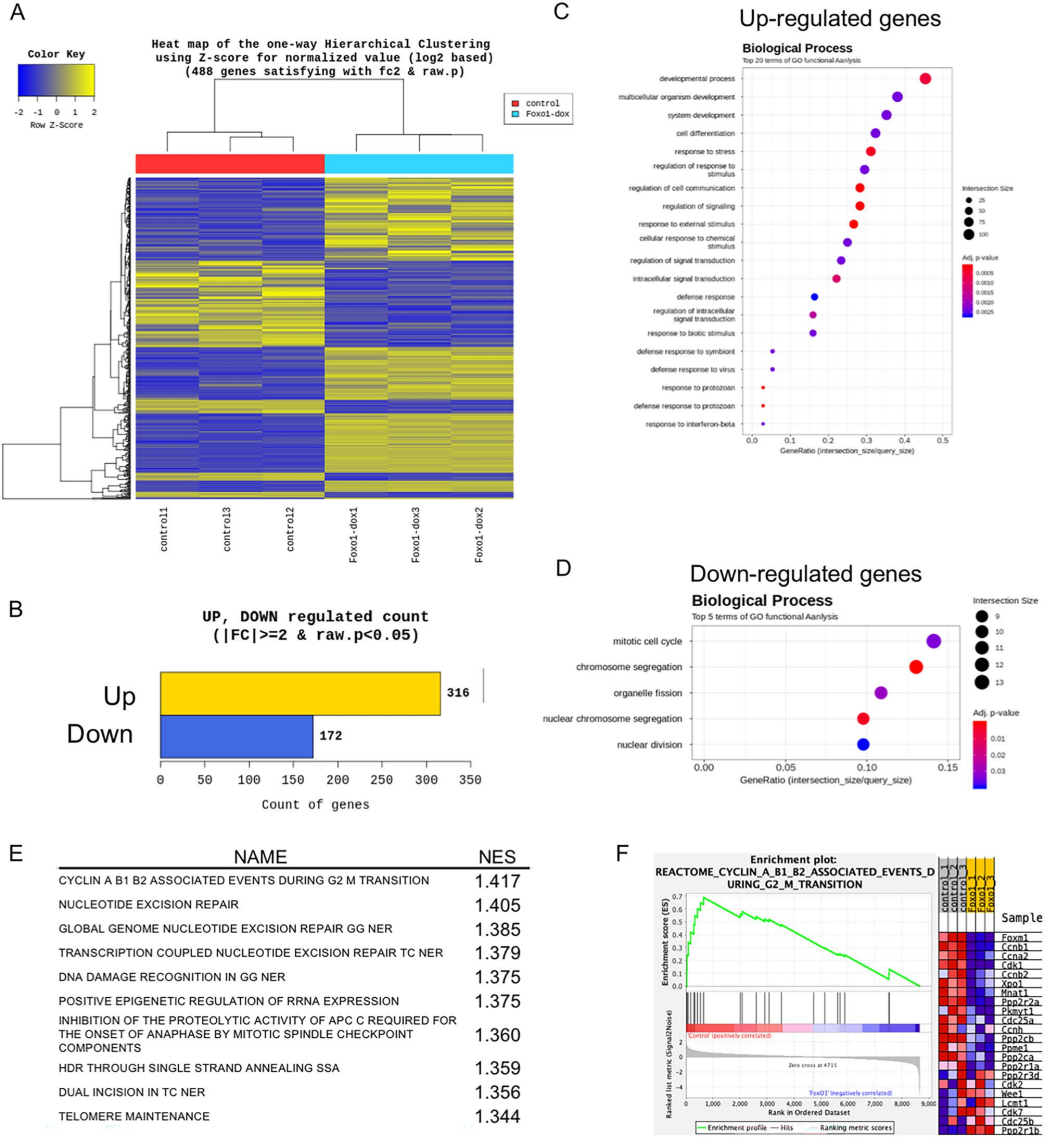
Transcriptome profiling of FoxO1-expressing ME cells. The RNA samples were prepared from ME^PB-FoxO1^ cells treated with and without Dox for 72 h. (**A**) Heatmap of differentially expressed genes (control vs. FoxO1). (**B**) The number of up- and down-regulated genes based on fold change of comparison pair (FoxO1/control ≥ 2, *P* < 0.05). (**C–F**) Enrichment of Gene Ontology terms for biological processes associated with up- (**C**) and down- (**D**) regulated genes. (**E,F**) Data from the gene set enriched analysis (GSEA). The top gene lists of normalized enrichment score (NES) are shown in (**E**). *P < 0.001. Enrichment plot of cyclin A B1 B2 associated events during G2 M transition are shown in (**F**). See also Supplementary Tables S2–S5.

To examine the transcriptional alterations in FoxO1-overexpressing ME cells, ME^PB-FoxO1^ cells with and without Dox treatment were compared by RNA-sequence analysis (Fig. 3A). A total of 316 genes were upregulated and 172 genes were downregulated in FoxO1-overexpressing cells (Fig. 3B, Tables S2, S4). Gene Ontology analysis revealed that the upregulated genes were related to developmental processes and cell differentiation (Fig. 3C, Table S3). In contrast, the expression of cell cycle-related genes was downregulated by FoxO1-overexpression (Fig. 3D, Table S5). Gene set enrichment analysis (GSEA) showed that FoxO1 regulates cyclin associated events (Fig. 3E) and listed genes such as Ccnb1, Ccna2, and Cdk1 which are inhibited by cyclin-dependent kinase inhibitors p21/CIP1/WAF1 and p27/Kip1 were downregulated by FoxO1 (Fig. 3F).

**Figure 4:**
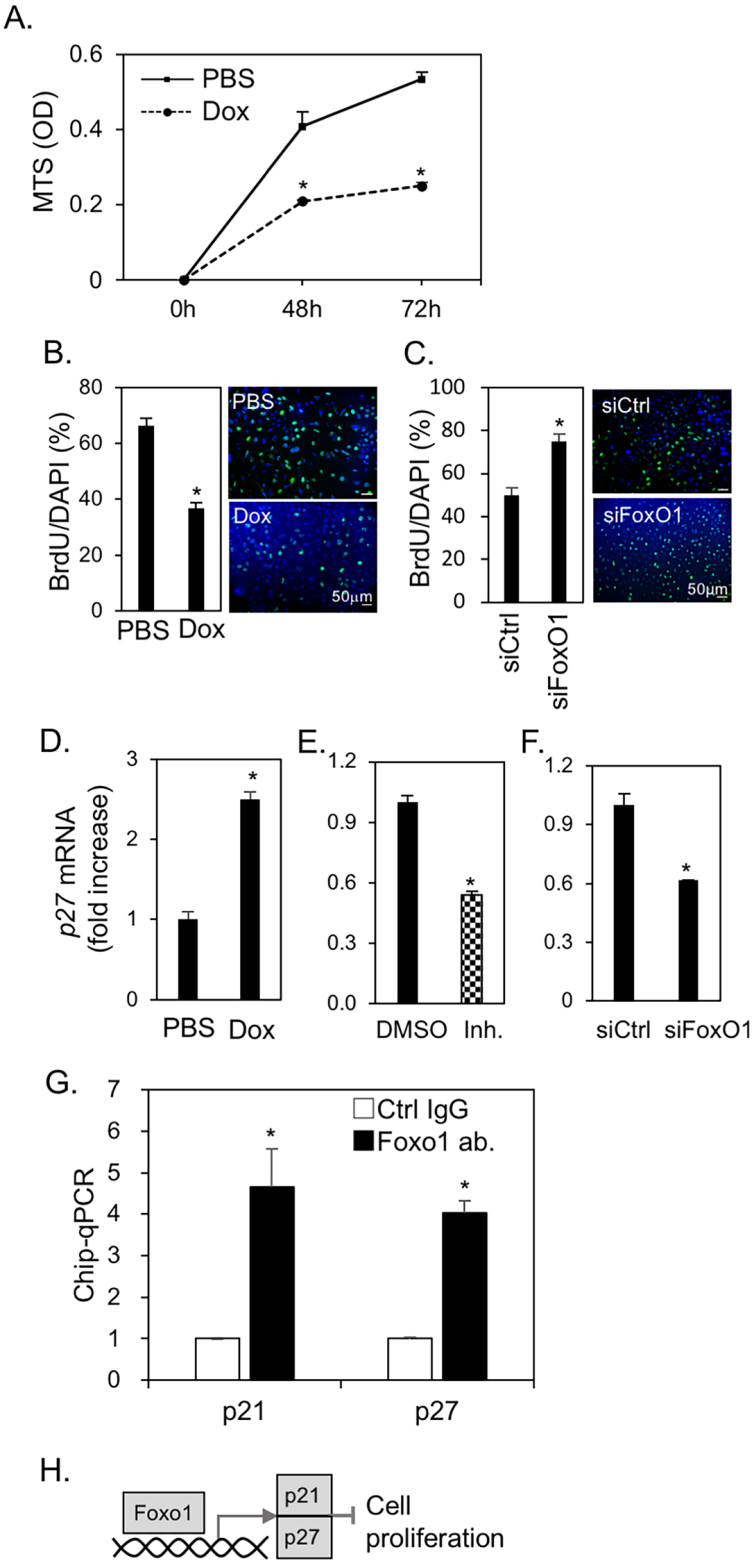
FoxO1 suppressed ME cell proliferation via cell cycle arrest. (**A**) Viability of ME^PB-FoxO1^ cells treated with and without Dox (2 µg/mL) at the indicated time-points. (**B,C**) Cell proliferation rates were measured by BrdU incorporation assay. BrdU positive/DAPI (%, left) with and without Dox (2 µg/mL) for 24 h (**B**) or with and without transfection of siRNA for FoxO1 for 48 h (**C**). Immunofluorescent images were showed on the right (BrdU; green, DAPI; blue). (**D–F**) Expression of *p27(KIP1)* in ME^PB-FoxO1^ cells. Cells were treated with and without Dox (2 µg/mL) (**D**), pretreated with and without FoxO1 inhibitor (Inh.; AS1842856, 1 μM) (**E**) and transfected with siRNA for FoxO1 (**F**) in the presence of Dox (2 µg/mL) for 48h. The expression data of *p21(CIP/WAF1)* were shown in Fig. S4. (**G**) Chromatin immunoprecipitation-quantitative real-time PCR (ChIP-qPCR) analysis of the DNA binding activity of FoxO1 in ME cells. DNA sample was prepared from ME^PB-FoxO1^ cells treated with Dox (2 µg/mL) for 72 h. The associated DNA at the promoter regions of *p21*^*CIP/WAF1*^ (− 1722 to − 1712) and *p27*^*KIP1*^ (− 1036 to − 1026), after incubation with FoxO1 antibody-conjugated protein G beads, were immunoprecipitated and analyzed by qPCR. *P < 0.05. n = 3. All data were representative of three independent experiments. (**H**) A schematic for FoxO1-induced cell growth inhibition. See also Supplementary Fig. S4.

Since FoxO1 downregulated cell cycle-related genes, cell viability and gene expression of the cyclin-dependent kinase inhibitors p21 and p27 were examined in ME^PB-FoxO1^ cells with and without Dox treatment. Dox-induced ME^PB-FoxO1^ cells showed lower proliferation than the controls by MTS assay (Fig. 4A). Dox-treated ME^PB-FoxO1^ cells (Fig. 4B) and gene silencing siFoxO1 (Fig. 4C) also decreased BrdU incorporation at 24 h and 48 h, respectively. These results suggest that cell proliferation is inhibited by FoxO1 and that the cell cycle is arrested. As expected, the expression of *p21* and *p27* expression was upregulated by Dox*,* whereas FoxO1 inhibitor suppressed the expression of *p21* and *p27* (Fig. 4D,E, Fig. S4)*.* siFoxO1 also decreased Dox-induced *p27* expression (Fig. 4F). Chip-qPCR revealed that FoxO1 bound to the promoter regions of *p21* (− 1722 to − 1712) and *p27* (− 1386 to − 1376), suggesting that FoxO1 induced cell cycle arrest through p21 and p27 (Fig. 4G). These results suggest that FoxO1 may directly induce the expression of *p21* and *p27* and induce cell arrest (Fig. 4H).

### FoxO1 and NF-κB contribute the expression of ectodysplasin A (Eda) and its receptor Eda2 receptor (Eda2r)

**Figure 5:**
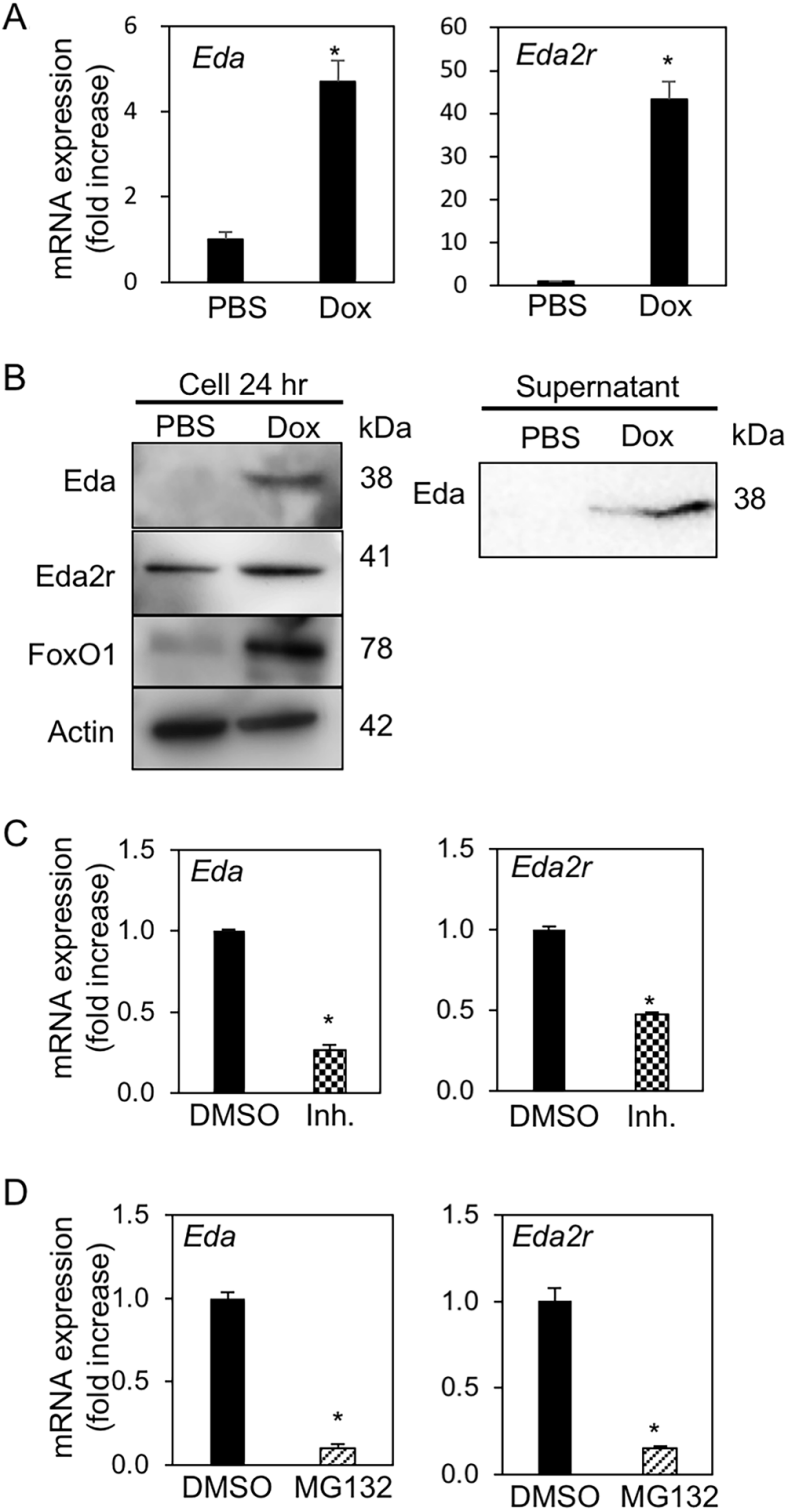
FoxO1 induced *Eda/Eda2r* expression in ME cells through NF-κB activation. (**A,B**) Gene expression (**A**) and immunoblotting (**B**) of Eda and Eda2r in ME^PB-FoxO1^ cells treated with and without Dox (2 µg/mL) for 24 h. (**C**) Expression of *Eda* and *Eda2r* in Dox-treated (2 µg/mL, 72 h) ME^PB-FoxO1^ cells with and without FoxO1 inhibitor (Inh.; AS1842856, 10 μM) pretreatment for 24 h. (**D**) Expression of *Eda* and *Eda2r* in Dox-treated (2 µg/mL, 72 h) ME^PB-FoxO1^ cells with and without NF-κB inhibitor (MG132, 20 μM) pretreatment for 6 h. *P < 0.05. n = 3. All data were representative of three independent experiments. See also Supplementary Fig. S5.

RNA sequence-based transcriptome analysis revealed that FoxO1 upregulated cell differentiation-related genes, such as *Eda* and its receptor, *Eda2r*, which are associated with branching morphogenesis of the salivary gland (Fig. 3C, Table S3). Dox- treated ME^PB-FoxO1^ cells showed increased mRNA expression of *Eda* and *Eda2r* (Fig. 5A), whereas treatment with the FoxO1 inhibitor as well as NF-κB inhibitor MG132 decreased Dox-induced *Eda* and *Eda2r* (Fig. 5C,D). Eda was also detected in the cell lysate and culture medium after Dox treatment (Fig. 5B), suggesting that the cleaved form of Eda was secreted. These results suggest that FoxO1 and NF-κB may synergistically contribute to Eda/Eda2r expression and Eda/Eda2r signaling might be upregulated by FoxO1.

### Salivary gland development is inhibited by the inhibitors of FoxO1 and NF-κB

**Figure 6:**
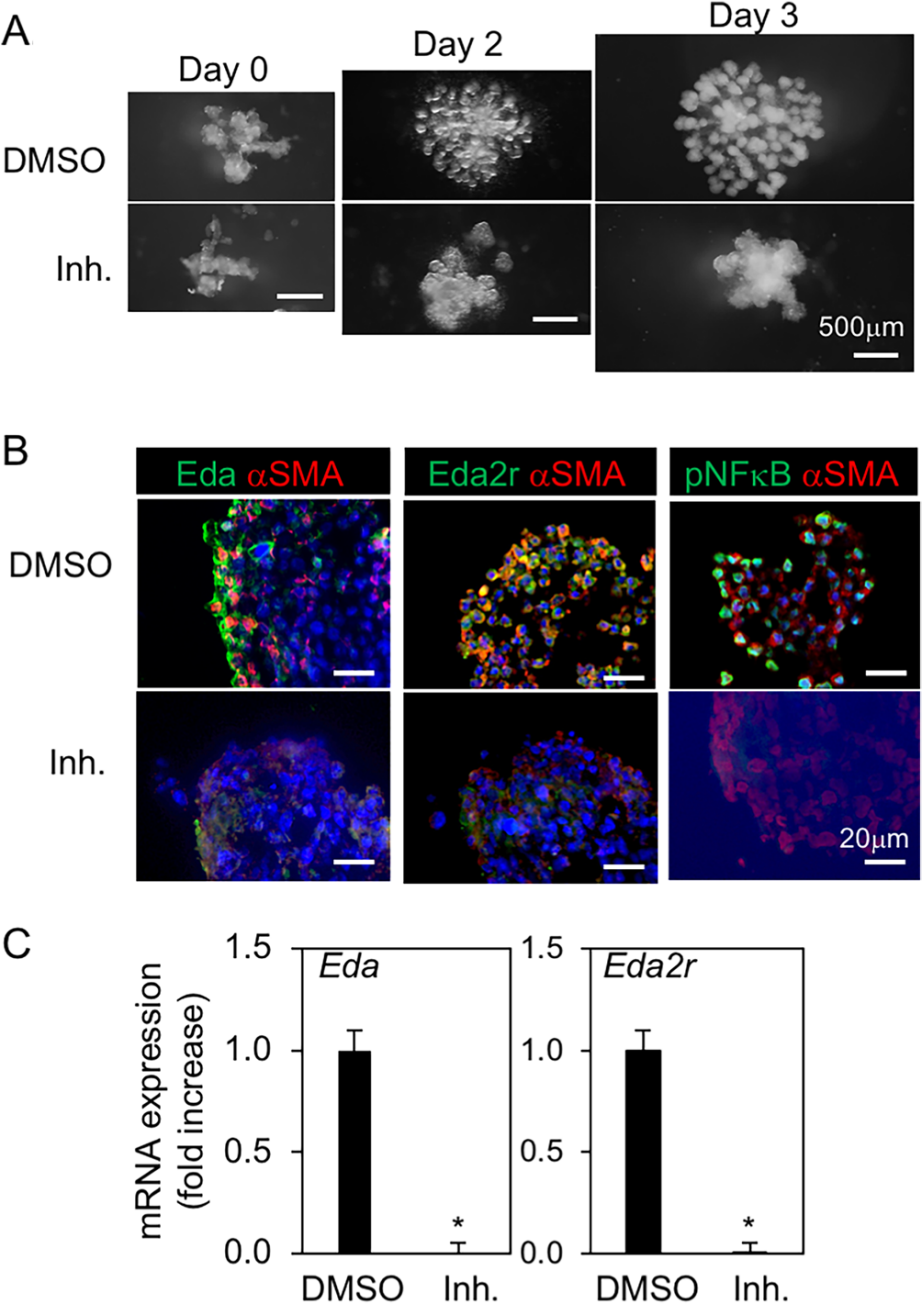
Inhibition of FoxO1 inhibited development of the primitive epithelium of SMG ex vivo. Epithelia of SMG rudiments on E14.5 (n = 4), were mounted in Matrigel drops and cultured in the presence of FGF1 and FGF7 with and without FoxO1 inhibitor (Inh.; AS1842856, 10 μM) for 3 days. (**A**) Phase contrast images. Bar = 500 μm. (**B**) The image of immnofluorescent of αSMA, Eda, Eda2r, and phospho-NF-κB after 3 days of culture. Nuclei were stained with DAPI. Bar = 20 μm. (**C**) Expression of *Eda* and *Eda2r* after 3 days of culture. *P < 0.05. All data were representative of three independent experiments. See also Supplementary Figs. S6 and S7.

**Figure 7:**
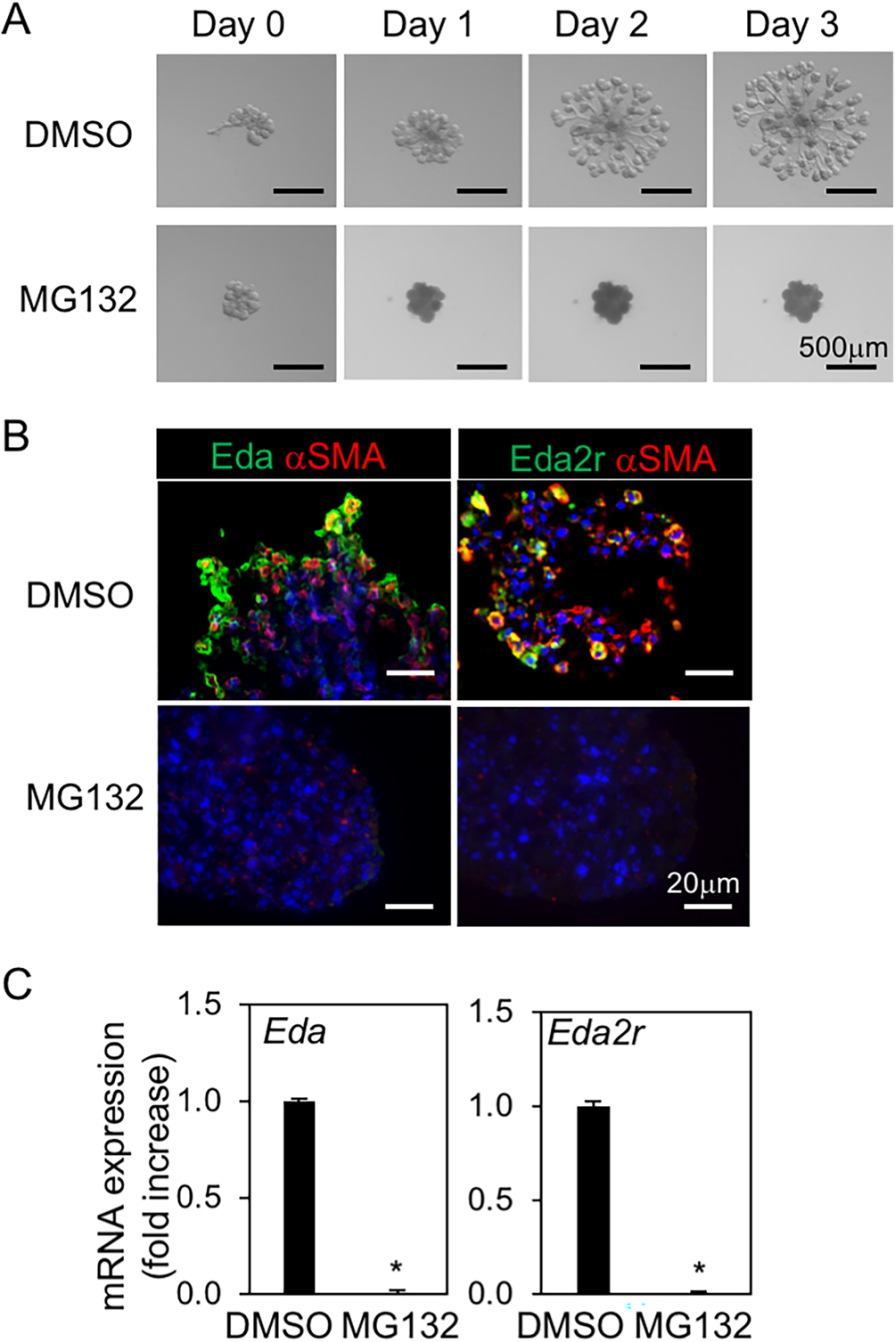
NF-κB was essential for developing the primitive epithelium of SMG with Eda/Eda2r expression ex vivo. Epithelia of SMG rudiments on E14.5 (n = 4) were mounted in Matrigel drops and cultured in the presence of FGF1 and FGF7 with and without NF-κB inhibitor (MG132, 20 μM) for 3 days. (**A**) Phase contrast image. Bar = 500 μm. (**B**) IF of αSMA, Eda, and Eda2r after 3 days of culture. Nuclei were stained with DAPI. Bar = 20 μm. (**C**) Expression of *Eda* and *Eda2r* after 3 days of culture. *P < 0.05. All data were representative of three independent experiments.

Subsequently, the SMG epithelium from the E14.5 SMG eliminated mesenchyme was cultured for 3 days with and without AS1842856. AS1842856 strongly inhibited the epithelial development (Fig. 6A). TUNEL staining were examining whether AS1842856 induced growth arrest of SMG primordial epithelium by apoptosis detected no signal (Fig. S6). Immunofluorescence of Eda was detected in the outer layer of endo buds and co-localized with αSMA-positive ME cells. Meanwhile, Eda2r was expressed in a wider range of endo buds, including ME cells at the E16 SMG primordium (Fig. S7). Similarly, Eda was expressed in the outer layer of endo buds, while Eda2r was expressed in a wider range of endo buds, including αSMA-expressing ME cells. In contrast, Eda, Eda2r, and αSMA expression was weakened in the SMG epithelia cultured with AS1842856 for 3 days (Fig. 6B). Consistent with protein expression, the gene expression of *Eda* and *Eda2r* in cultured tissue were strongly inhibited by AS1842856 (Fig. 6C). Interestingly, phospho-NF-κB immunofluorescence was also observed in the outer region of control end buds co-expressing αSMA, similar to the E16 SMG primordium, whereas it was completely inhibited by AS1842856 (Fig. 6B, Fig. S7). These results suggest that FoxO1 dependent NF-κB and Eda/Eda2r activation in ME cells located at outer layer of endo buds contributes to salivary branching morphogenesis. To examine whether FoxO1 regulated to the expression of Eda and Eda2r via NF-κB, SMG epithelium at E14.5 were also cultured for 3 days with and without the NF-κB inhibitor (MG132) (Fig. 7A). MG132 completely prevented the development of SMG epithelium by inhibiting the mRNA and protein levels of Eda/Eda2r (Fig. 7A–C). These results suggest that FoxO1 and NF-κB may contribute to Eda/Eda2r signaling-dependent SMG development.

## Discussion

In the present study, we examined the role of transcriptional factor FoxO1 in ME cells and elucidated that FoxO1 restricted ME cell overgrowth via cyclin-dependent kinase inhibitors and FoxO1 and NF-κB may contribute to Eda/Eda2r signaling-dependent SMG development. Further direct investigation is needed since excess levels of constitutively active FoxO1 may significantly alter the normal cell response and the perceived activity of the transcription factor. The isolation of myoepithelial cells was previously hampered owing to a lack of specific markers. However, it has been reported that myoepithelial cells can be isolated in mammary glands by flow cytometry using the combination of EpCAM and CD49f^12,38^. According to these results, we expected that ME cells could be identified as CD49f-high and EpCAM-low cells. Indeed, these cells expressed ME cell-marker, αSMA. Additionally, we used a transgenic Mhy11CreERtdTfloxed mouse model for ME cell isolation in previous studies^11,12^. We found that 98% of tdT cells were CD49f-high and EpCAM-low cells. Thus, we confirmed that CD49f-high and EpCAM-low cells were ME cells in salivary glands.

FoxO1 is negatively regulated by the PI3K-AKT signaling pathway. AKT-mediated FoxO1 phosphorylation at T24, S253, and S316 in mice^39^ and at T24, S256, and S319 in humans^40^ induces proteasomal degradation of cytoplasmic FoxO1^41^. PhosphoFoxO1‐S319 was detected in the minor salivary gland epithelia of patients with Sjögren’s syndrome, which is a severe hypofunction of the salivary glands^35,37^, suggesting that inactivation of FoxO1 induces tissue degradation, especially in secretory units such as acini and ME cells. Downregulation of FoxO1 in the salivary gland in Sjögren’s syndrome causes a direct reduction of AQP5-expressing cells^36^. AQP5 is expressed not only in mature acini but also in ME and acinic precursors^5^. The involvement of ME cells in acinar cell differentiation and proliferation is interesting. ME cells can regulate acinar differentiation via FGF7 secretion owing to histological localization of myoepithelial cells around acini^42^. FoxO1 is predominantly expressed in ME cells at both the embryonic and adult stages; however, it remains unclear whether FoxO1 is involved in the differentiation of acinar cells. Tissue damage caused by FoxO1 inactivation in the salivary glands of patients with Sjögren’s syndrome also suggests that FoxO1 may regulate autoimmune responses, epigenetic modifications, cell death, and inflammation^35–37,43,44^. FoxO binds to extended and condensed chromatin structures and performs diverse physiological functions^45^. Here, FoxO1-mediated expression of *p21*/*p27* inhibited ME cell proliferation. Transcriptome analysis revealed that FoxO1 also induced retinoblastoma (Rb), a tumor suppressor that negatively regulates the G1/S transition in the mitotic cell cycle. pRb-dependent chromatin remodeling suppresses the transcription of cell type-specific transcription factors, including MyoD, myogenin, and C/EBP^46^. The gene sets related to cell differentiation were top-ranked in the GO analysis of the upregulated genes in FoxO1-expressing ME cells, including Eda/Eda2r. Therefore, FoxO1 might regulate ME cell proliferation and differentiation.

Eda is a tumor necrosis factor family transmembrane protein and its receptor Edar triggers a signaling cascade of NF-κB and induces downstream target genes required for the development of hair, teeth, and exocrine glands^47–49^. X-linked hypohidrotic ectodermal dysplasia (XHED), caused by mutations in EDA or EDAR, is characterized by hypoplasia and dysfunction of ectodermal-derived tissues, such as the skin, teeth, sweat glands, and salivary glands^47^. The critical role of Eda/Edar in salivary glands was examined using Eda-null mice (Tabby) and Eda- overexpressing transgenic mice driven by the cytokeratin 14 promoter^49–52^. Eda strictly controlled branching morphogenesis by regulating the number of end buds in embryos and ductal and acinar structures in the adult stage of these mice^50,51^. The downstream of Eda/Edar/NF-κB and potential cross talk with Wnt, Shh signaling pathway in salivary gland have already been reported^2,49,50^; however, the signaling crosstalk with FoxO1 and Eda/Eda2r in the salivary glands remains unclear. Eda2r (XEDAR) is differ from Edar, whose ligands are Eda-A2 and Eda-A1, respectively. Eda-A2 is an isoform of Eda-A1 and is only two bases shorter^48^. Eda2r mutations are reported in XHED patients^48,53^. Overexpression of Eda-A2 and Eda2r were reported in salivary epithelium from the patients of Sjögren’s syndrome^54^. However, it remains unclear whether it has the same effect as Eda/Edar. Further study is required to determine the effect of Eda2r on salivary gland development. Interstingly, FoxO1 and NF-κB-binding sites were predicted in the promoter region of Eda/Eda2r in silico. Examination the predicted FoxO1-binding sites ([T/G/C]TGTTTA) and NF-κB-biding sites (GGG[A/G]A[T/A]T[T/C][C/T][C/T]) in the Eda2r promoter (up to − 3 kbp) in silico identified one of FoxO1- (position at -126) and three in NF-κB- (position at − 650, − 2318, − 2748) binding sites by FIMO^55^. On the other hand, FoxO1 binding sites (position at − 4031, − 3935, − 3134) were predicted in Eda promoter (up to − 5 kbp) although there are two FoxO1-binding sites and one NF-κB-biding sites were predicted between exon 1 and exon 3 (no exon 2 in mouse Eda^56^). Direct expression of Eda/Eda2r requires further investigation, but FoxO1 and NF-κB may contribute to their expression. Both FoxO1 and NF-κB signaling are required for salivary gland development^52^, suggesting that FoxO1 and NF-κB may contribute synergistically to Eda/Eda2r signaling and salivary branching morphogenesis. While Foxo1 arrests the cell cycle, FoxO1 inhibitors suppressed salivary gland primordium growth without rescuing cell cycle arrest. A discrepancy arises with FoxO1 inhibitors against salivary gland primordial development. Cell cycle arrest and development by FoxO1 were separately observed in this study. Inhibition of cell proliferation by FoxO1 were examined in vitro using ME cells from adult mice. However, salivary gland development was examined in embryonic organ cultures. Therefore, FoxO1 may play different roles in ME cells during the embryonic and adult stage. Another possibility is that FoxO1-mediated cell arrest may occur in ME cells in the organ culture and that FoxO1 inhibitor may cause proliferation of ME cells. However, FoxO1 inhibitor downregulates αSMA, Eda, and Eda2r in these cells, although it is unknown why excess FoxO1 expression did not affect αSMA expression. Thus, ME cells may be induced to dedifferentiate and lose normal functions. In addition, Eda/Eda2r signaling induced by FoxO1 is important for paracrine action on endo bud cells in salivary gland development. Thus, failure of salivary gland primordial epithelial development caused by FoxO1 inhibitor may result from disruption of the supply of Eda to endo bud cells. Cell cycle arrest promotes cell differentiation^45^. Therefore, FoxO1 may restrict ME cell overgrowth and subsequently induce cell differentiation during the development stage or in the tumor environments. However, the precise mechanism of action of ME cells in salivary gland development remains unclear.

FoxO1-null mice are embryonically lethal at E11 due to impaired angiogenesis^29,57,58^, whereas FoxO3- and FoxO4-null mice are viable^59^. Although FoxO1, FoxO3, and FoxO4 are highly related homologs, FoxO1 deficiency cannot be compensated for by other FoxO family members. Therefore, examination of FoxO1-specific function in each tissue type is needed. Inactivation of FoxO1 induces salivary gland hypofunction in Sjögren’s syndrome, as described above. FoxO1 is also involved in tumor development, suppression, and progression in various types of tissues, such as soft tissue sarcoma, acute myeloid leukemia, breast cancer, hepatocellular cancer, gastric cancer, and B-cell lymphoma^60^. In salivary glands, PAX3:FOXO1 chimeric fusion proteins have been detected in rhabdomyosarcoma^61^, while PLAGL1:FOXO1 fusion proteins have been detected in pleomorphic adenoma^62^. However, there are limited reports on FoxO1 expression in salivary gland tumors. Dysregulation of FoxO1 signaling may affect neoplastic changes in ME cells but further investigations are required to understand ME cell biology.

In summary, we found that FoxO1 was predominantly expressed in ME cells. FoxO1 limited abnormal ME cell proliferation via p21/p27, cyclin-dependent kinase inhibitors, and salivary epithelial development through Eda/Eda2r signaling. FoxO1 dysregulation in the abnormal condition of salivary glands may induce cell diversity by regulating ME transdifferentiation.

## Experimental procedures

### Mice

The experimental protocol was approved by the Institutional Animal Care and Use Committee of Showa University (Approval Nos. 15018, 14040, 13031, and 18002). All mouse strains used in this study have been previously described^12^. Myh11-CreER^T2^/tdTomato^fl/fl^ mice (CAG-tdTomato mice, JR#007914; Myh11-CreER^T2^ mice, JR#019079; Jackson Laboratory, Bar Harbor, ME) were used. Trp53-null (p53^−/−^) mice^63^ were provided by the RIKEN BioResource Center (RBRC01361; Tsukuba, Ibaraki, Japan).

### Reagents

AS1842856 (1 or 10 μM; #S8222), which reduces FoxO1-mediated transactivation by directly binding to active FoxO1 without affecting its transcription and protein expression (FoxO3a, 3%; FoxO4, 20%)^64,65^, and MG132 (20 μM; #S2619), a proteasome inhibitor with the potential to inhibit NF-κB^66^, were purchased from Selleck Chemicals (Houston, TX).

### Cell culture

A mouse ME cell line was established from p53-deficient mice as described previously^12,38^. p53-deficient mice used in this study have only one mutation in the p53 gene and possess many advantages to establish primary culture cells compared with gene transfer of the simian virus 40 (SV40) T antigen. The p53 gene does not affect normal development but is frequently mutated, allowing these cell lines to keep up to passage 10^12,38^. Briefly, the mouse submandibular glands were minced, digested with collagenase I (750 U/mL; Wako) and hyaluronidase (500 U/mL; Sigma) for 45 min at 37 °C, and further dissociated into single cell suspensions by TrypLE Express (Gibco). The isolated cells (1 × 10^6^ cells/mL) were stained with fluorescent conjugated antibodies at 1:500 dilution for 30 min on ice and analyzed by flow cytometry (Cell Sorter SH800, Sony Biotechnology, Tokyo, Japan)^12,38^. The EpCAM-low and CD49f-high cell fraction was collected as ME cells. p53 did not thought to affect normal ME cell population analyzed accordingly to our previous study^12,38^. The cells were maintained in Keratinocyte-SFM medium supplemented with epidermal growth factor and bovine pituitary extract (Gibco, Gaithersburg, MD) under the condition of 5% CO_2_ at 37 °C. To achieve stable expression of mouse FoxO1 (NM_019739.3) in ME cells (ME^PB-FoxO1^), FoxO1-ADA (#35640; Addgene, Cambridge, MA) was cloned into PB-TAC-ERN (#80475; Addgene) according to the manufacturer's instructions. FoxO1-ADA is a constitutively active FoxO1 mutant containing the following amino acid substitutions: T24A, S253D, and S316A^25^. Subsequently, the cells (2 × 10^5^) were co-transfected with PBase (1 μg) and PB-TAC-ERN-FoxO1-ADA (1 μg) using a Neon Transfection System (1200 V, 20 ms, 2 pulses; Invitrogen) for electroporation. A stable line was established by drug selection using G418 (400 μg/mL, Sigma-Aldrich, St. Louis, MO). The PiggyBac plasmid carried a tetracycline response element to drive doxycycline (Dox) induction (2 µg/mL; LKT Labs, St. Paul, MN).

### FoxO1 small interfering RNA (siRNA)

Cells (2 × 10^5^) were transfected with FoxO1 Mouse siRNA Oligo Duplex (#SR427332/SR418715, OriGene) and control siRNA duplex (#SR30002, OriGene) at a final concentration of 100 nmol/L using Lipofectamine RNAiMax reagent (Invitrogen, #13778-100) without antibiotics according to the manufacturer’s instructions. After 24 h incubation, the media was replaced with fresh medium and cells were harvested for further analysis after indicated further incubation.

### Transcriptome analysis

Total RNA was extracted after lysis of the samples in 1 mL TRIzol reagent (Invitrogen), followed by phase separation with 0.2 mL chloroform (Wako, Osaka, Japan) and RNA precipitation with 0.5 mL isopropyl alcohol (Wako). The total RNA (1 μg) with A260/280 ratio > 1.8 was reverse-transcribed to cDNA using SuperScript VILO (Invitrogen). Quantitative real-time RT-PCR was performed using SYBR Green I dye (Applied Biosystems, Waltham, MA) and analyzed using a 7500 Fast Real-Time PCR System (Applied Biosystems). Quantification of the samples was performed according to the threshold cycle using the ΔΔCt method. The experiments were repeated thrice. The primer sequences are listed in Table S1. The values presented in the graphs represent the mean ± SD.

For mRNA-sequence analysis, total RNA was extracted using an RNeasy Plus Mini Kit (Qiagen, Valencia, CA). Library preparation and sequencing were performed by Macrogen Japan Corp. (Tokyo, Japan). Briefly, the quality of RNA was measured using an Agilent Bioanalyzer (RIN > 7). mRNA-focused libraries from total RNA (1 μg, > 20 ng/mL) were prepared with a TruSeq stranded mRNA LT Sample Prep Kit (Illumina), DNA sequencing was performed on a NovaSeq 6000 (ver. 1.7.0) using NovaSeq 6000 S4 Reagent Kit v1.5. All libraries were run at pair-ends (2 × 100 bp). Zero count filtering were performed for data analysis. For cDNA mapping, mm10 was used as a reference genome. The mapping data statistics were obtained from HISAT2, which is known to handle spliced read mapping through Bowtie2 aligner. After the read mapping, StringTie was used for transcript assembly (annotation: NCBI_108). The expression profile was calculated for each sample and transcript/gene as read count, FPKM (Fragment per Kilobase of transcript per Million mapped reads) and TPM (Transcripts Per Million Kilobase). Differentially Expressed Genes (DEG) analysis was performed on a comparison pair (Foxo1-dox_vs_control) as requested using edgeR. The results showed that 488 genes which satisfied |fc| ≥ 2 & exactTest raw P-value < 0.05 conditions in the comparison pair. Sequence data were also analyzed using the CLC Genomics Workbench ver.23 (Filgen, Nagoya, Japan).

### Immunofluorescence

SMGs obtained from MyhCreER:tdTomato^fl/fl^ mice or SMG organ cultured tissues were fixed in 4% paraformaldehyde (Wako) at 4 °C for 1 h, followed by cryopreservation with sucrose (Wako). The cryosections (4 μm) were prepared and subjected to immunofluorescence using anti-mouse antibodies against FoxO1 (1:50; #2880; Cell Signaling Technology, Danvers, MA) and E-cad (1:100; #610181; BD Biosciences, Franklin Lakes, NJ), Eda (1:100; #PA5-72840; Invitrogen), Eda2r (1:100; #BS-7111R; Bioss), and phospho-NF-κB p65 (1:100; #3033; Cell signaling) overnight at 4 °C, followed by incubation with Alexa Fluor secondary antibody (1:200; Invitrogen) for 1 h at room temperature. Nuclei were counter-stained with DAPI (Dojindo, Kumamoto, Japan). Images were captured using a BZ-9000 fluorescence microscope (Keyence, Tokyo, Japan). FoxO1-positive areas were counted using a hybrid cell count application (BZ-H4C, Keyence) in BZ-X Analyzer software (BZ-H4A, Keyence).

### Western blotting

Cells were lysed using the RIPA Lysis Buffer System supplemented with a protease inhibitor cocktail, sodium orthovanadate, and PMSF (Santa Cruz Biotechnology, Dallas, TX). Total protein (20 μg) samples were loaded into each well of SDS-PAGE gel (10%) for separation by electrophoresis and transferred to a PVDF membrane (0.2 μm; GE HealthCare, Chicago, IL). Membranes were blocked for 1 h with TBS-Tween 20 containing 5% ECL prime blocking reagent (Cytiva, Tokyo, Japan) and incubated overnight at 4 °C with primary antibodies against FoxO1 (1:1000; #2880; Cell Signaling Technology), αSMA (1:1000; #ab7817; Abcam), Krt14 (1:1000; #ab7800; Abcam), Krt5 (1:1,000; #ab52635; Abcam), β-actin (1:1000; #A5060; Sigma-Aldrich), Eda (1:500; #PA5-72840; Invitrogen), Eda2r (1:1000; #BS-7111R; Bioss), phospho-NF-κB p65 (1:1000; #3033; Cell signaling), and NF-κB p65 (1:1000; #8242; Cell signaling). After washing with TBS-T, the membranes were incubated with secondary antibodies (1:5000) against anti-rabbit IgG (#NA934; Cytiva) and anti-mouse IgG (#NA391; Cytiva). Immunoreactivity was detected using ECL Prime western blotting detection reagent (Cytiva) and photographed using an Amersham Imager 600 (Cytiva).

### Measurement of FoxO1 reporter activity

Firefly luciferase vector (pGL4 vector; 1 μg) containing three copies of either the FoxO1 DBE-binding cassette, daf-16 (TTGTTTA) or mutant daf-16 (TTGCTTA)^67^ and Renilla luciferase (phRG-TK vector; 0.1 μg) were co-transfected into ME^PB-FoxO1^ cells (3.5 × 10^4^ cells/well in a 96-well plate) using Lipofectamine LTX Reagent with Plus Reagent (Invitrogen). The FoxO1 inhibitor was added at the indicated concentrations 24 h before Dox (2 μg/mL) induction for 48 h. Luciferase assays were performed using the Dual-Glo Luciferase Assay System (Promega, Madison, WI), according to the manufacturer’s instructions. Firefly luciferase activity was normalized to the Renilla luciferase activity. Data are expressed relative to the controls. All values are presented as the means ± SD. Three independent experiments were performed in triplicate.

### Chip assay

ME^PB-FoxO1^ cells were treated with 10% formaldehyde neutral buffer solution for 10 min to cross-link proteins and chromatin. The reaction was stopped by incubating with 2.5 M glycine for 5 min. The cells were washed twice with cold PBS containing a protease inhibitor. Cells were then resuspended in 1 mL ChIP lysis buffer [1 M HEPES–KOH (pH 7.5), 5 M NaCl, 0.5 M EDTA, 10% NP40, 10% Triton-X100] for 10 min on ice and centrifuged at 2500 rpm to pellet the nuclei. The cell nuclei were resuspended in nuclei lysis buffer [2 M Tris–HCl (pH 8.0), 0.5 M EDTA, 0.5 M EGTA, 5 M NaCl] and then subjected to sonication six times for 30 s at 3-min intervals. Purified chromatin was analyzed on a 1% agarose gel to determine the shearing efficiency. For control, normal IgG was used instead of FoxO1 antibody (#2880; Cell Signaling Technology). The amount of chromatin precipitated by the indicated antibodies was determined using qPCR. The ChIP-qPCR primers were as follows: p21^CIP1^ promoter (− 1722 to − 1712: TCCGTTCAAACTAAGACTCCA;TAGCGCTTGCCTAACATGTAT) and p27^KIP1^ promoter (− 1036 to − 1026: TAGATGTTGGTAATACCGTGG; CGCCTTTATACCCTTATGTTC)^68^.

The relative amount (% recovery) of the control region of immunoprecipitated DNA compared to the input DNA was calculated as follows: % recovery = 2^[(Ct input−log2(X)) −Ct immunoprecipitation sample]^ × 100, where (X) represents the dilution factor (fold) of the input sample.

### MTS assay and cell proliferation assay

ME^PB-FoxO1^ cells were seeded in 96-well plates at a density of 5 × 10^3^ cells/well. After 1 day, Dox (2 μg/mL) was added to each well. Cell proliferation was evaluated using the Cell Count Reagent SF (Nacalai Tesque Inc., Kyoto, Japan) after 1, 2, or 3 days. For, BrdU incorporation assay, cells were incubated in the 10 μM BrdU labeling solution for 2 h at 37 °C in a CO2 incubator. BrdU labeled cells were fixed with 4% PFA and permeabilized and detected with primary antibodies against BrdU (1:100, # clone: BMC9318;Roche) for 1 h, followed by standard immunocytochemistry (ICC) protocols.

### Organ culture of primordial SMGs

Organ cultures of primordial submandibular glands have been described previously^69,70^. Briefly, SMGs were dissected from E14.5 mice, digested with DNase I (Sigma-Aldrich) and dispase (Corning), and the mesenchyme around the epithelium was removed. The epithelium was embedded in 20 μL of Matrigel (#356231; Corning) and cultured in DMEM/F12HAM (Gibco) containing BSA (A9418; Sigma-Aldrich), ITS (Gibco), rhFGF1 (#064-04781; Wako), and rhFGF7 (#119099661; Wako).

### Statistical analysis

Unpaired Student’s t-test was used to compare two independent groups. Statistical significance was set at P < 0.05.

## Supplementary Information

Supplementary Information.

## Data Availability

All data generated or analyzed during this study are included in this published article and its Supplementary Information files.

## Abbreviations

Eda: Ectodysplasin A
HED: X-linked hypohidrotic ectodermal dysplasia
ME: Myoepithelial
EpCAM: Epithelial cell adhesion molecule
CD49f: Alpha 6 integrin
αSMA: Alpha smooth muscle actin
dox: Doxycycline
E-cad: E-cadherin
tdT: TdTomato fluorescence
SMG: Submandibular glands
