# Author Correction: Transcription factor FoxO1 regulates myoepithelial cell diversity and growth

**DOI:** 10.1038/s41598-025-04535-x

**Published:** 2025-06-17

**Authors:** Rino Tokumasu, Rika Yasuhara, Seya Kang, Takahiro Funatsu, Kenji Mishima

**Affiliations:** 1 Division of Pathology, Department of Oral Diagnostic Sciences, School of Dentistry, Showa University, Tokyo, 142-8555 Japan; 2 Division of Dentistry for Persons With Disabilities, Department of Perioperative Medicine, Graduate School of Dentistry, Showa University, Tokyo, 142-8555 Japan; 3 Division of Dentistry for Persons With Disabilities, Department of Perioperative Medicine, School of Dentistry, Showa University, Tokyo, 142-8555 Japan; 4 Department of Pediatric Dentistry, School of Dentistry, Showa University, Tokyo, 142-8555 Japan

Correction to: *Scientific Reports* 10.1038/s41598-024-51619-1, published online 11 January 2024

The original version of this Article contained an error in Fig. 4B, where, as a result of an error during figure assembly, a duplicate figure had been uploaded.

**Fig. 4:**
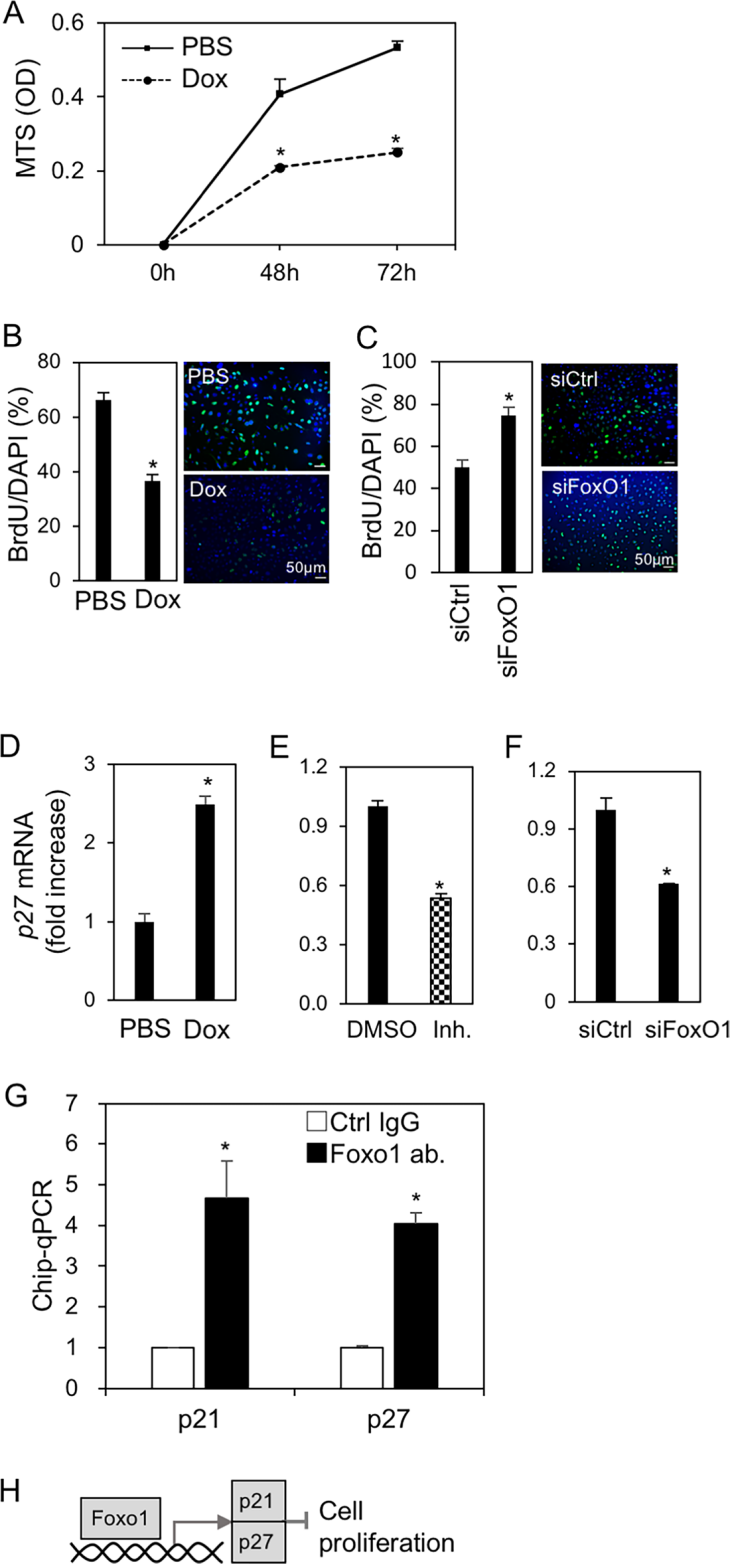
FoxO1 suppressed ME cell proliferation via cell cycle arrest. (**A**) Viability of ME^PB-FoxO1^ cells treated with and without Dox (2 µg/mL) at the indicated time-points. (**B,C**) Cell proliferation rates were measured by BrdU incorporation assay. BrdU positive/DAPI (%, left) with and without Dox (2 µg/mL) for 24 h (**B**) or with and without transfection of siRNA for FoxO1 for 48 h (**C**). Immunofluorescent images were showed on the right (BrdU; green, DAPI; blue). (**D–F**) Expression of *p27(KIP1)* in ME^PB-FoxO1^ cells. Cells were treated with and without Dox (2 µg/mL) (**D**), pretreated with and without FoxO1 inhibitor (Inh.; AS1842856, 1 μM) (**E**) and transfected with siRNA for FoxO1 (**F**) in the presence of Dox (2 µg/mL) for 48h. The expression data of *p21(CIP/WAF1)* were shown in Fig. S4. (**G**) Chromatin immunoprecipitation-quantitative real-time PCR (ChIP-qPCR) analysis of the DNA binding activity of FoxO1 in ME cells. DNA sample was prepared from ME^PB-FoxO1^ cells treated with Dox (2 µg/mL) for 72 h. The associated DNA at the promoter regions of *p21*^*CIP/WAF1*^ (− 1722 to − 1712) and *p27*^*KIP1*^ (− 1036 to − 1026), after incubation with FoxO1 antibody-conjugated protein G beads, were immunoprecipitated and analyzed by qPCR. *P < 0.05. n = 3. All data were representative of three independent experiments. (**H**) A schematic for FoxO1-induced cell growth inhibition. See also Supplementary Fig. S4.

The original Article has been corrected.

