# Supplementary material for: Transcription factor FoxO1 regulates myoepithelial cell diversity and growth
Source: Sci Rep. 2024 Jan 11;14:1069. doi: 10.1038/s41598-024-51619-1 (PMC10784559; doi:10.1038/s41598-024-51619-1)
Supplement: Supplementary file 1 — Supplementary Information. [file 41598_2024_51619_MOESM1_ESM.pdf]

Transcription factor FoxO1 regulates myoepithelial cell diversity and growth

Rino Tokumasu[1, 2], Rika Yasuhara[1*], Seya Kang[3], Takahiro Funatsu[4], Kenji Mishima[1*]

[1] Division of Pathology, Department of Oral Diagnostic Sciences, School of Dentistry, Showa University, Tokyo, Japan, 142-8555.
[2] Division of Dentistry for Persons with Disabilities, Department of Perioperative Medicine, Graduate School of Dentistry, Showa University, Tokyo, Japan, 142-8555.
[3] Division of Dentistry for Persons with Disabilities, Department of Perioperative Medicine, School of Dentistry, Showa University, Tokyo, Japan, 142-8555.
[4] Department of Pediatric Dentistry, School of Dentistry, Showa University, Tokyo, Japan, 142-8555.

Supplemental Table S1. Primer pairs size (bp)

GAPDH	forward	5'-AAGCCCATCACCATCTTCCAGGAG-3'	310
	reverse	5'-ATGAGCCCTTCCACAATGCCAAAG-3'	
αSMA	forward	5’-GGAGAAGCCCAGCCAGTCGC-3’	117
	reverse	5’-AGCCGGCCTTACAGAGCCCA-3’	
FoxO1	forward	5'-CTACGAGTGGATGGTGAAGAGC-3'	138
	reverse	5'-CCAGTTCCTTCATTCTGCACTCG-3'	
p21	forward	5'-TCGCTGTCTTGCACTCTGGTGT-3'	124
	reverse	5'-CCAATCTGCGCTTGGAGTGATAG-3'	
p27	forward	5'-AGCAGTGTCCAGGGATGAGGAA-3'	133
	reverse	5'-TTCTTGGGCGTCTGCTCCACAG-3'	
Eda	forward	5'-TCAACAGCAGCCTTTGGAACCG-3'	132
	reverse	5'-CGAACACGCCTACTTTCCTCTTC-3'	
Eda2r	forward	5'-AAGCAGACTCCTTCTTCCGAGG-3'	105
	reverse	5'-CACCAGTGCAACAAGTGTGGCT-3'	

Table S2. Top 30 Up-regulated gene list (FoxO1TPM mean>5, Control TPM mean>1). Related to Fig.3B.

Gene	Description	FoxO1 /Control	FoxO1 mean	Control mean
Foxo1	forkhead box O1	21.66	595.41	27.49
Tmsb15l	thymosin beta 15b like	13.07	24.04	1.84
Ghr	growth hormone receptor	5.79	40.23	6.95
Ifi209	interferon activated gene 209	4.62	8.72	1.89
Gnai1	guanine nucleotide binding protein (G protein), alpha inhibiting 1	3.96	21.79	5.50
Ifi44	interferon-induced protein 44	3.90	8.10	2.08
H1f2	histone cluster 1, H1c	3.51	31.36	8.95
Bdh2	3-hydroxybutyrate dehydrogenase, type 2	3.48	5.74	1.65
Sdcbp2	syndecan binding protein (syntenin) 2	3.36	18.66	5.56
Pik3ip1	phosphoinositide-3-kinase interacting protein 1	3.27	10.78	3.30
Cpq	carboxypeptidase Q	3.20	7.56	2.36
Nfe2l3	nuclear factor, erythroid derived 2, like 3	3.17	44.27	13.98
Rb1	RB transcriptional corepressor 1	3.15	11.12	3.53
Tmem140	transmembrane protein 140	3.14	12.86	4.09
Cytip	cytohesin 1 interacting protein	3.02	7.84	2.60
Igfbp2	insulin-like growth factor binding protein 2	2.98	54.15	18.18
Irf7	interferon regulatory factor 7	2.93	5.10	1.74
Efhd1	EF hand domain containing 1	2.92	41.17	14.11
Eda2r	ectodysplasin A2 receptor	2.86	6.41	2.24
Usp18	ubiquitin specific peptidase 18	2.84	5.71	2.01
Entpd3	ectonucleoside triphosphate diphosphohydrolase 3	2.80	10.43	3.73
Nipal1	NIPA-like domain containing 1	2.78	7.51	2.70
Itm2a	integral membrane protein 2A	2.76	17.16	6.22
Plaat3	phospholipase A and acyltransferase 3	2.73	27.94	10.25
Cyp4v3	cytochrome P450, family 4, subfamily v, polypeptide 3	2.72	8.35	3.07
Prss12	protease, serine 12 neurotrypsin (motopsin)	2.69	5.69	2.11
Pamr1	peptidase domain containing associated with muscle regeneration 1	2.62	6.65	2.54
Mmp13	matrix metallopeptidase 13	2.61	24.41	9.34
Isg15	ISG15 ubiquitin-like modifier	2.56	21.26	8.29
Klf15	Kruppel-like factor 15	2.56	19.69	7.70

Table S3. GO term: Developmental process(FoxO1 TPM mean >2.5, Control TPM mean>1). Related to Fig.3C.

Gene	Fold Change	Control_1	Control_2	Control_3	FoxO1_1	FoxO1_2	FoxO1_3
Foxo1	21.66	26.77	27.46	28.25	647.11	588.69	550.42
Ghr	5.79	7.43	6.51	6.91	40.68	39.14	40.86
Bdh2	3.48	1.94	1.40	1.61	5.78	5.09	6.36
Cpq	3.20	2.47	2.15	2.46	7.92	7.70	7.05
Rb1	3.15	3.20	3.61	3.79	10.89	11.92	10.56
Arhgdib	3.04	1.13	1.24	1.36	3.20	3.92	4.21
Igfbp2	2.98	19.75	16.20	18.60	47.83	54.63	59.99
Efhd1	2.92	13.05	15.67	13.61	42.04	40.01	41.46
Grk5	2.88	1.90	1.34	1.85	4.91	5.05	4.66
Eda2r	2.86	2.29	2.03	2.40	5.81	7.23	6.20
Itm2a	2.76	6.75	6.08	5.83	15.61	17.26	18.60
Wdpcp	2.74	1.15	1.72	0.87	3.23	3.56	3.42
Plaat3	2.73	10.14	10.26	10.35	26.83	27.99	29.00
Mmp13	2.61	10.00	9.19	8.84	26.54	24.74	21.96
Isg15	2.56	8.41	7.87	8.60	17.61	26.09	20.09
Klf15	2.56	8.49	7.13	7.47	20.37	19.46	19.25
Eda	2.47	0.99	1.06	1.04	3.08	2.55	2.01
Lratd1	2.42	1.90	1.40	1.37	3.97	3.54	3.81
Chn1	2.42	2.33	2.47	2.40	6.20	5.21	6.05
Slit2	2.41	1.30	1.33	1.52	3.77	3.06	3.16
Ctla2a	2.36	3.95	5.97	4.98	11.64	10.28	13.30
Nrp1	2.25	2.89	2.83	3.31	6.57	7.34	6.41
Cebpd	2.23	5.44	4.91	4.29	11.60	10.06	10.97
Arrdc3	2.13	2.18	2.28	2.13	4.85	4.71	4.50
Arhgap18	2.12	1.39	1.09	1.39	2.84	3.30	2.07
Btbd2	2.09	4.33	3.95	4.87	9.23	9.34	8.93
Eya1	2.08	1.75	1.45	1.44	3.45	3.05	3.16
Alcam	2.04	2.64	2.77	2.53	5.79	5.67	4.74
Uchl1	2.04	8.66	7.03	7.40	16.23	15.05	15.83

Table S4. Down-regulated gene list (FoxO1 TPM mean<10, Control TPM mean>5). Related to Fig.3B.

Gene	Description	FoxO1 /Control	FoxO1 mean	Control mean
Txnip	thioredoxin interacting protein	-3.86	2.84	10.97
Cpa4	carboxypeptidase A4	-3.57	1.48	5.27
Mmp12	matrix metallopeptidase 12	-3.15	6.74	21.23
Cyba	cytochrome b-245, alpha polypeptide	-3.00	2.29	6.88
Ulbp1	UL16 binding protein 1	-2.67	4.54	12.13
Sprr1b	small proline-rich protein 1B	-2.50	2.79	6.97
Snora73b	small nucleolar RNA, H/ACA box 73b	-2.49	2.06	5.13
Snord68	small nucleolar RNA, C/D box 68	-2.48	2.69	6.67
LOC115488444	U6 spliceosomal RNA	-2.46	2.23	5.48
Shcbp1	Shc SH2-domain binding protein 1	-2.40	2.20	5.27
Adamts8	a disintegrin-like and metallopeptidase (reprolysin type) with thrombospondin type 1 motif, 8	-2.33	7.18	16.76
LOC115489087	small nucleolar RNA SNORD31	-2.28	4.40	10.03
Dlgap5	DLG associated protein 5	-2.24	5.51	12.34
Kifc1	kinesin family member C1	-2.23	7.78	17.37
LOC115489726	uncharacterized LOC115489726	-2.22	3.09	6.87
LOC115490091	U6 spliceosomal RNA	-2.21	2.87	6.34
Nusap1	nucleolar and spindle associated protein 1	-2.20	5.17	11.36
Sprr2j-ps	small proline-rich protein 2J, pseudogene	-2.17	2.88	6.26
Cp	ceruloplasmin	-2.14	9.68	20.71
Mki67	antigen identified by monoclonal antibody Ki 67	-2.13	5.64	12.02
Snord14a	small nucleolar RNA, C/D box 14A	-2.11	3.26	6.89
Hmmr	hyaluronan mediated motility receptor (RHAMM)	-2.10	2.67	5.62
Snord35a	small nucleolar RNA, C/D box 35A	-2.07	2.63	5.43
Mirt1	myocardial infarction associated transcript 1	-2.03	2.97	6.03
Kif18b	kinesin family member 18B	-1.99	2.87	5.72
Cenpk	centromere protein K	-1.99	3.86	7.67
Tubb3	tubulin, beta 3 class III	-1.99	9.25	18.36

Table S5. GO term: Mitotic cell cycle. Related to Fig.3D.

Gene	Fold change	Control_1	Control_2	Control_3	FoxO1_1	FoxO1_2	FoxO1_3
Dact1	-2.65	0.7	1.2	1.2	0.3	0.3	0.6
Kifc1	-2.23	14.2	19.4	18.5	7.4	8.8	7.2
Nusap1	-2.20	11.1	11.5	11.6	4.0	5.3	6.2
Mki67	-2.13	10.7	12.3	13.0	5.1	6.3	5.6
Ticrr	-2.12	1.4	1.8	1.8	0.7	0.8	0.9
Ska1	-2.12	0.5	0.7	0.5	0.2	0.3	0.3
Cdc6	-2.06	2.9	2.3	2.7	1.3	1.1	1.5
Cenpf	-2.03	1.8	2.7	2.5	1.1	1.2	1.2
Sapcd2	-2.07	0.9	1.6	1.6	0.6	0.9	0.4
Eme1	-1.94	1.0	1.4	1.0	0.6	0.8	0.3
Cenpk	-1.99	7.7	7.6	7.7	4.0	3.6	4.0
Kif18b	-1.99	5.2	6.4	5.5	2.8	2.8	3.0
Tubb3	-1.99	16.1	19.2	19.8	8.5	10.5	8.7

↑ Tokumasu et al. Supplementary Fig. S1, related to Fig. 1

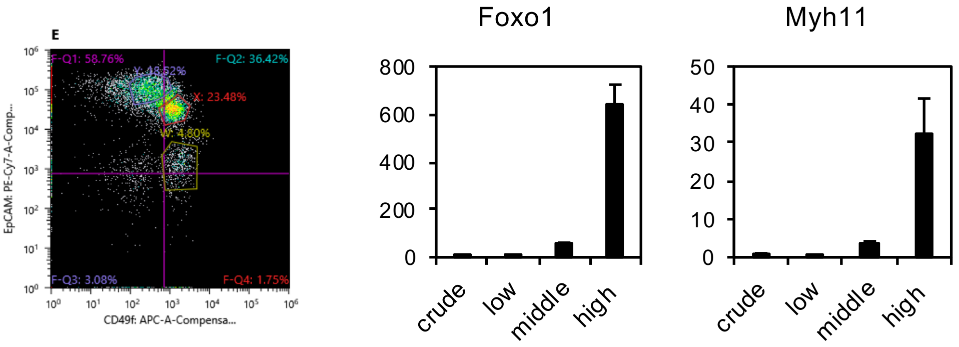

Figure S1. FoxO1 expressed in Myh11-positive cells, related to Fig. 1.
Scatter plot of CD49f (x-axis) and EpCAM (y-axis). The expression of Myh11 was high in EpCAM^{low}CD49f^{high}-cells (W), and that of FoxO1 also was high.

↑ Tokumasu et al. Supplementary Fig. S2, Fig. 2E and 2I

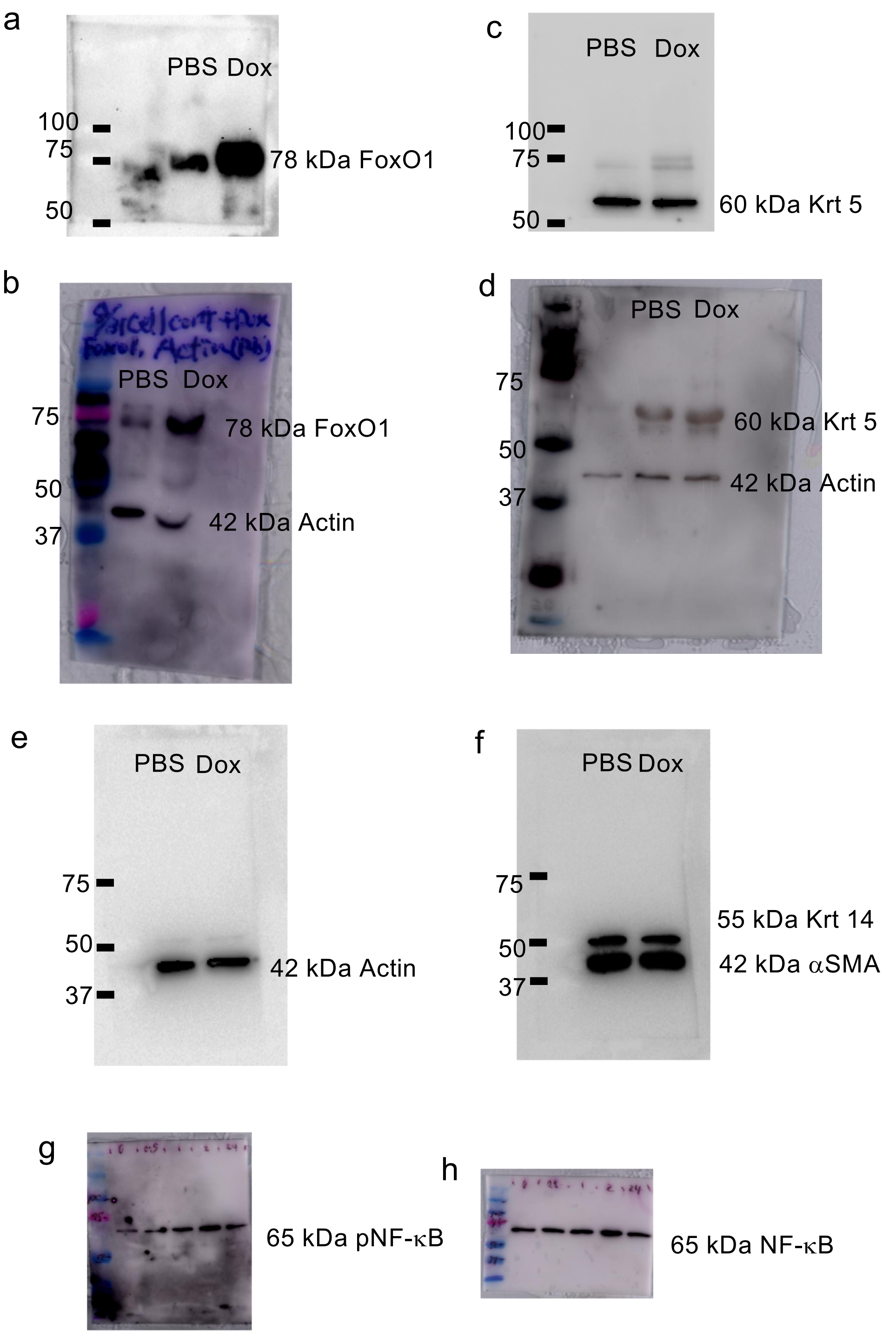

Figure S2. Raw data of Western Blot results, related to Fig. 2E, 2I.
(a-f) Immunoblotting for FoxO1 (a, b), Krt5 (c, d), β-actin (e), αSMA (f), and Krt14 (f) in $ME^{PB\text{-}FoxO1}$ cells treated with and without Dox for 72 h, related to Fig. 2E. (b, d) The replicates of entire membranes of a. and c. showed in b. and d. because the membranes of a and c were separated by 50 kDa. (g-h)Immunoblotting for NF-κB/p65 and phospho-NF-κB/p65 in $ME^{PB\text{-}FoxO1}$ cells treated with and without Dox at the indicated time-points. The full uncropped blot from Western Blot results, related to Fig. 2I.

↑ Tokumasu et al. Supplementary Fig. S3, related to Fig. 2F

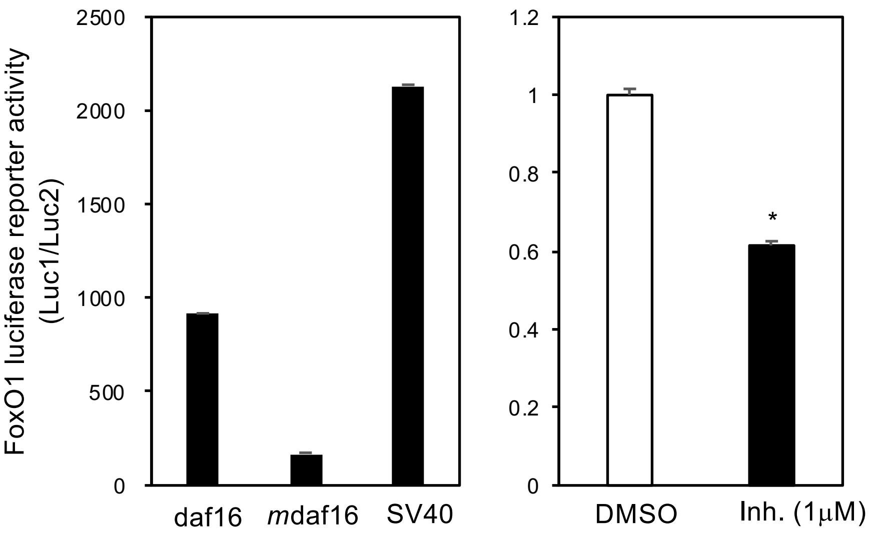

Figure S3. FoxO1 reporter activity, related to Fig, 2F.
Endogenous FoxO1 expression decreased in the presence of FoxO1 inhibitor (Inh; AS1842856, 1μM) (right). Base level of FoxO1 luciferase reporter activity was detected. SV40 (pGL4.13 luc2/SV40, #E6681, Promega), reporter vector was used for positive control (left). *P<0.05.

↑ Tokumasu et al. Supplementary Fig. S4, related to Fig. 4C-4E

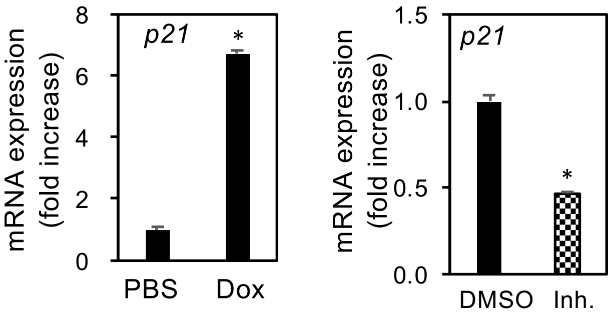

Figure S4. Gene expression of p21, related to Fig.4C-4E.
Expression of *p21(CIP/WAF1)* in $ME^{PB\text{-}FoxO1}$ cells treated with and without Dox (2 µg/mL) for 72 h (left), and with and without FoxO1 inhibitor (Inh.; AS1842856, 1 µM) pretreatment for 24 h (right). *P<0.05.

↑ Tokumasu et al. Supplementary Fig. S5, related to Fig. 5B

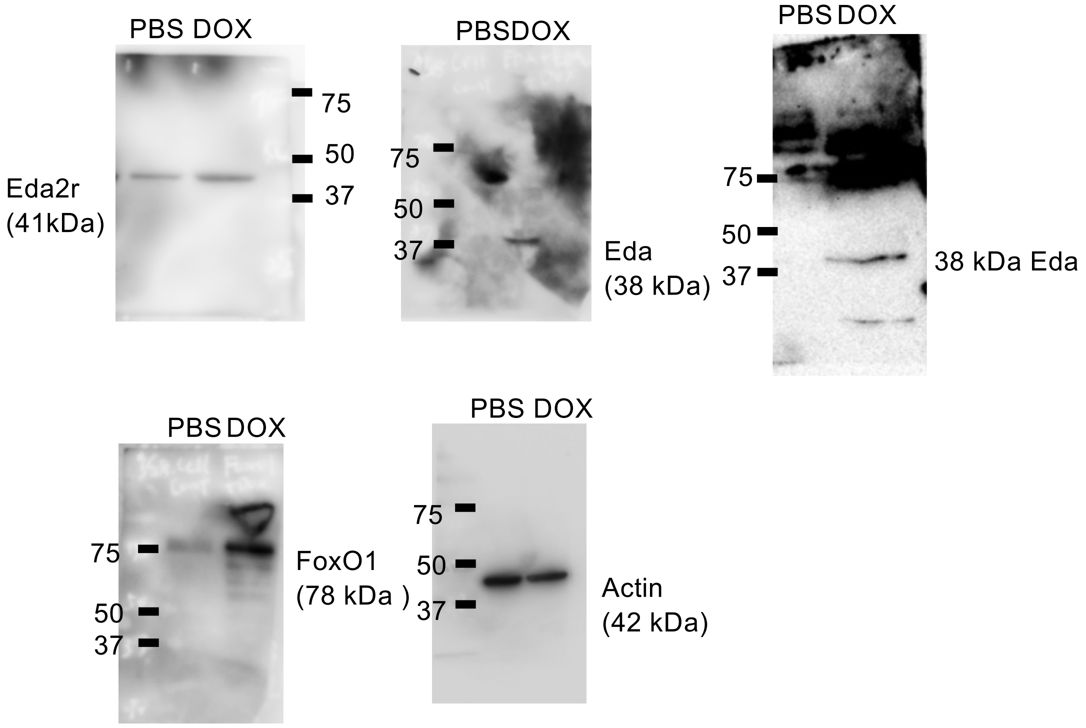

Figure S5. Raw data of Western Blot results, related to Fig, 5B.
(Fig5. B) Immunoblotting of Eda and Eda2r in ME$^{PB\text{-}FoxO1}$ cells treated with and without Dox (2 µg/mL) for 24 h. The full uncropped blot from Western Blot results.

↑ Tokumasu et al. Supplementary Fig. S6, related to Fig. 6 and 7

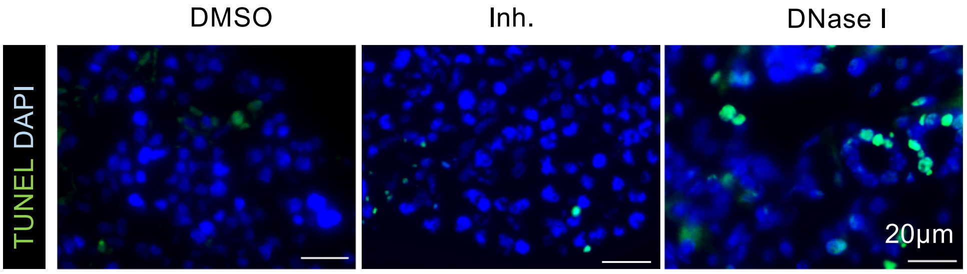

Figure. S6. TUNEL Assay to examine apoptosis after FoxO1 inhibitor treatment in organ culture of primordial SMGs, related to Fig. 6 and 7.

The SMG epithelium from E14.5 SMGs eliminated mesenchyme was cultured for 1 day with and without AS1842856 (10 µM). The SMG epithelium was fixed in 4 % paraformaldehyde at 4 ℃ for O/N, followed by cryopreservation with sucrose. The cryosections were 4 µm. We used In Situ Cell Death Detection Kit, Fluorescein (#11684795910, Roche). Fix tissue section with 4% paraformaldehyde in PBS, pH 7.4, for 20 minutes at room temperature. After wash in PBS, the tissues were permeabilized in 0.1% Triton X-100 for 2 minutes on ice. Then add 50 µl TUNEL reaction mixture on sample and incubate for 60 minutes at 37 ℃ in a humidified atmosphere in the dark. Rinse slides 3 times with PBS. Nuclei were stained with DAPI . Images were captured using a BZ- 9000 fluorescence microscope. No TUNEL signal were observed in the control (left) and AS1842856 (10µM) additions (middle). Tissue sections treated with DNase I (10µg/ml) showed positive TUNEL images (right). bar=20µm.

↑ Tokumasu et al. Supplementary Fig. S7, related to Fig. 6 and 7

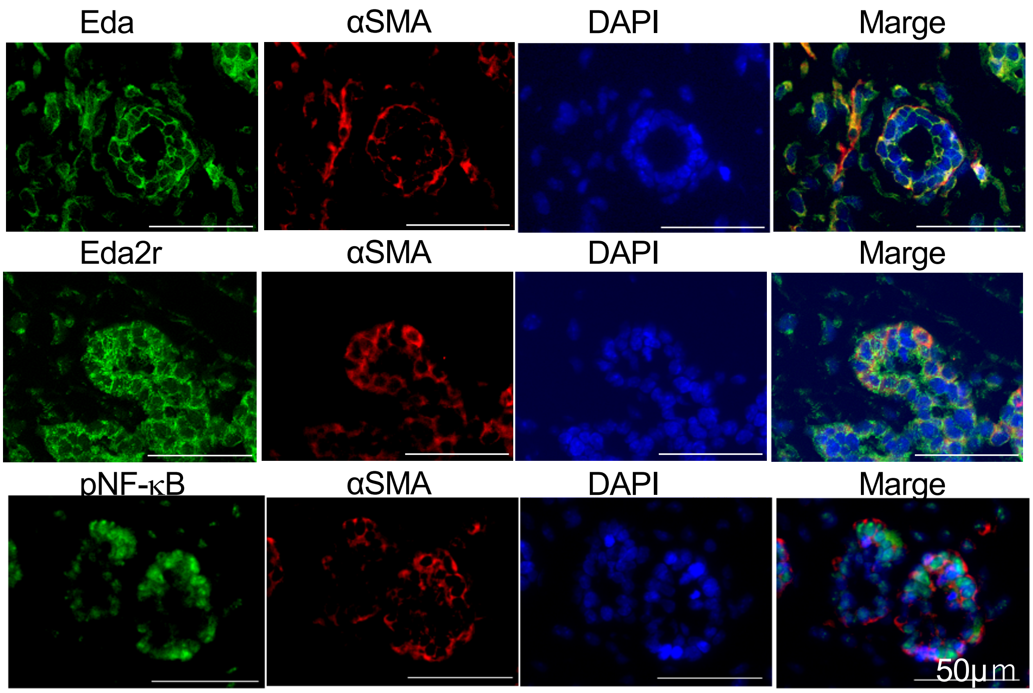

Figure S7. Localization of phospho-NF-κB, Eda, and Eda2r in ME cells of SMGs, related to Fig. 6 and 7. IF of αSMA, Eda, Eda2r, and phospho-NF-κB (pNF-κB) in SMGs from E16 WT mice. Nuclei were stained with DAPI. bar =50 μm.
